# Characterization of site-specific glycosylation of secreted proteins associated with multi-drug resistance of gastric cancer

**DOI:** 10.18632/oncotarget.8287

**Published:** 2016-03-23

**Authors:** Jian Wu, Hongqiang Qin, Ting Li, Kai Cheng, Jiaqiang Dong, Miaomiao Tian, Na Chai, Hao Guo, Jinjing Li, Xin You, Mingming Dong, Mingliang Ye, Yongzhan Nie, Hanfa Zou, Daiming Fan

**Affiliations:** ^1^ State Key Laboratory of Cancer Biology and Xijing Hospital of Digestive Diseases, Fourth Military Medical University, Xi'an 710032, China; ^2^ Key Laboratory of Separation Sciences for Analytical Chemistry, National Chromatographic R & A Center, Dalian Institute of Chemical Physics, Chinese Academy of Sciences, Dalian 116023, China

**Keywords:** gastric cancer, secretome analysis, chemoresistance, glycoprotein, site-specific glycosylation

## Abstract

Multi-drug resistance (MDR) remains a great obstacle to effective chemotherapy for gastric cancer. A number of secreted glycoproteins have been reported to be involved in the development of MDR in gastric cancer. However, whether glycosylation of secreted glycoproteins changes during MDR of gastric cancer is unclear. Our present work manifested that N-glycosites and site-specific glycoforms of secreted proteins in drug-resistant cell lines were distinctly different from those in the parental cell line for the first time. Further characterization highlighted the significance of some aberrantly glycosylated secretory proteins in MDR, suggesting that manipulating the glycosylation of specific glycoproteins could be a potential target for overcoming multi-drug resistance in gastric cancer.

## INTRODUCTION

Gastric cancer (GC) ranks the second most commonly diagnosed malignancy and the second most lethal cancer in China [1]. Despite the great improvements in diagnostic and therapeutic techniques of gastric cancer, the 5-year overall survival of GC patients still remains unsatisfactory. And, the emergence of multi-drug resistance (MDR) is largely responsible for the dismal long term survival of GC patients [2].

To date, there have been extensive studies on the mechanism of MDR. And, a number of molecules such as ATP-binding cassette (ABC) transporters have been found to be involved in the development of MDR [3]. To explore the underlying mechanisms of MDR in GC, the multi-drug resistant cell lines, SGC7901/ADR and SGC7901/VCR, were derived from human gastric adenocarcinoma cell line SGC7901 by stepwise selection with adriamycin and vincristine respectively in our lab. Based on these drug-resistant cell models, we have discovered a series of microRNAs (miR-19a/b, miR-508-5p, miR-27b, miR-129-5p) and proteins (HIF-1α, MGr1-Ag/37LRP, CCNG1) that could modulate the development of MDR in gastric cancer [4–8]. Besides, a growing number of researches concerning the role of tumor microenvironment in MDR declare that macrophages, T cells, fibroblasts and ECM (extracellular matrix) within tumor microenvironment could contribute to MDR of multiple cancers by direct and indirect interactions with tumor cells [9–12]. As critical components of the tumor microenvironment, secreted proteins of tumor cells also have been demonstrated to be greatly involved in chemoresistance. Targeted therapy with BRAF, ALK or EGFR kinase inhibitors induces a complex network of secreted factors in human melanoma and lung adenocarcinoma cells, and this therapy-induced secretome stimulates the outgrowth and dissemination of drug-resistant cancer cells and promotes the survival of drug-sensitive cancer cells [13]. Besides, extracellular vesicles derived from drug-resistant cancer cells could confer drug resistance on drug-sensitive cancer cells [14, 15]. Secreted clusterin (sCLU) was reported to contribute to MDR of several cancers such as osteosarcoma and hepatocellular carcinoma [16, 17]. In another example, secreted sonic hedgehog was proved to be of great significance in the development of MDR in multiple myeloma [18]. As for gastric cancer, Yuan et al. claimed that WNT6, a secreted glycoprotein, promoted multi-drug resistance of gastric cancer [19], and Cho et al. indicated that VEGF-C secretion contributed to chemoresistance of gastric cancer to cisplatin [20]. More importantly, the proteomic research accomplished by Huang et al. reported that secreted proteins were quite different between drug-sensitive and drug-resistant gastric cancer cell lines [21].

As one of the most abundant post-translational modifications (PTMs), glycosylation exercises great influences on not only structures but also functions of glycoproteins. Our previous study demonstrated that P-gp, a pivotal player in MDR, was aberrantly glycosylated in MDR gastric cancer cell lines and glycosylation at Asn99 residue might greatly affect its function in MDR [22]. Intriguingly, recent researches indicated that glycomic alterations of glycoproteins exerted enormous effects on chemoresistance of cancers. Lattova et al. verified the correlations between N-glycosylation variations and the efficacy of chemotherapies in breast cancer cells [23, 24] and discovered that the emergence of galactosylated or biantennary fucosylated glycans might be associated with chemoresistance. Besides, Nakano et al. observed that α 2–6 sialylation of N-glycans decreased with acquired resistance to desoxyepothilone B in leukemia cells [25]. Yet, the former researches mainly focused on glycosites or glycan structures. But, there are many glycosites in one glycoprotein, as well as many glycans at the same glycosite, which makes the glycosylation much more complex. Intact glycopeptides simultaneously contain the sequence of peptides, glycosites as well as glycan structures corresponding to the sites. And there have been several reports about the determination of site-specific glycoforms by MS, including N- and O-linked glycosylation [26–28]. In our previous work, site-specific glycoforms of total cell proteins were determined by combining de-glycopeptide and intact glycopeptide analysis [29]. Up to now, however, the identification and quantification of intact glycopeptides on large-scale remains a huge challenge, especially for the analysis of glycosylation of low abundance secreted glycoproteins.

Herein, we fabricated a platform for characterizing the glycosylation heterogeneity of secreted proteins in our MDR models, which included concentration of secreted proteins, enrichment of intact glycopeptides and determination of site-specific glycoforms. Additionally, the glycosites and site-specific glycoforms of secreted proteins were quantified, which comprised glycosite occupancy in protein as well as different glycan structures on the same glycosite. Totally, 1033 glycosites were identified from secreted proteins of SGC7901, SGC7901/ADR and SGC7901/VCR, including 240 sites with different glycosylation occupancy. A total of 2222 site-specific glycoforms were determined, and 499 site-specific glycoforms on 151 sites were significantly different between SGC7901 and its MDR sublines. To the best of our knowledge, this is the first large-scale analysis of site-specific glycoforms of secreted proteins in gastric cancer. Consequently, the comprehensive characterization of site-specific glycosylation could help us better understand the sophisticated mechanism of MDR in gastric cancer, thus promoting the discovery of feasible biomarkers for MDR and improving the efficiency of chemotherapy.

## RESULTS

### Generation of secretome datasets from 3 gastric cancer cell lines

**Figure 1:**
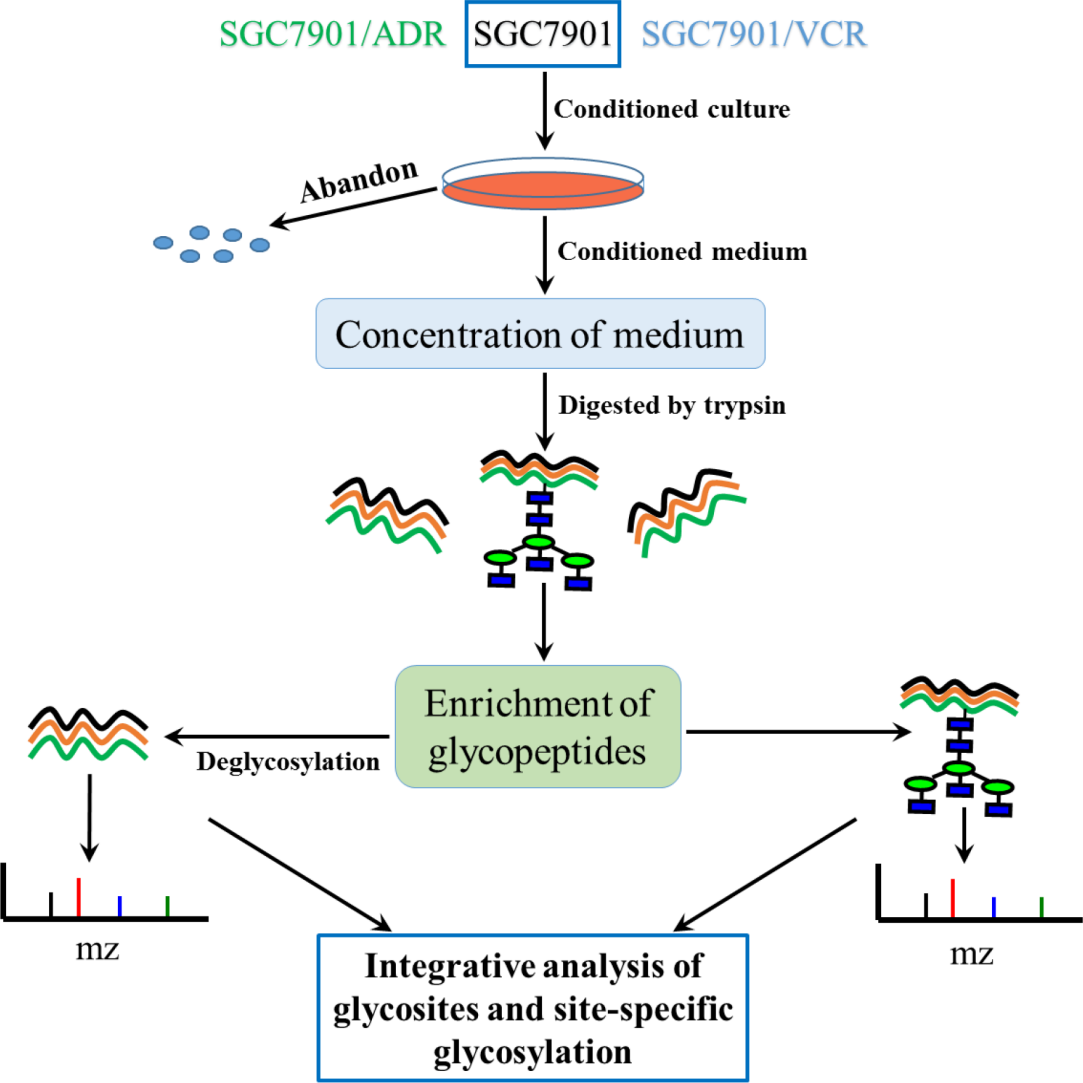
Workflow for the characterization of site-specific glycosylation of glycoproteins in secretomes of SGC7901, SGC7901/ADR and SGC7901/VCR

As stated above, the secretome of tumor cells played important roles in the process of acquired chemoresistance. Consequently, the analysis of secreted glycoproteins on proteome level could help us understand the specific mechanism of drug resistance. Due to the very low abundance, the analysis of secreted glycoproteins is still a big challenge, especially for gastric cancer cells and tissues. Thus, the secreted proteins need to be concentrated before analysis. As shown in Figure 1, the serum-free culture medium was collected and then concentrated by ultra filters (UF). The secreted proteins with low abundance could be effectively concentrated, and the salts in medium could be removed at the same time. Additionally, the glycopeptides were enriched by using hydrophilic interaction liquid chromatography (HILIC), which could retain the glycan structures linked to glycosites with high specificity. For the determination of site-specific glycoforms, the information of backbone peptide sequence, glycosite location and glycan structures was obtained by analysis of de-glycopeptides and intact glycopeptides, respectively.

**Figure 2:**
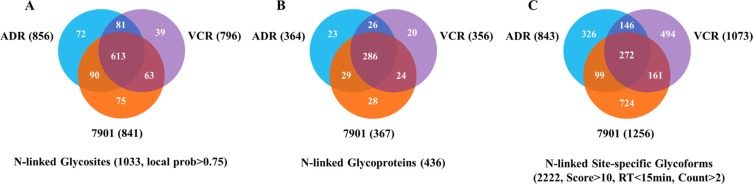
Venn diagram for numbers of N-glycosites, N-glycoproteins and site-specific glycoforms identified in SGC7901 and its MDR derivates 7901: SGC7901; ADR: SGC7901/ADR; VCR: SGC7901/VCR.

Generally speaking, a total of 1033 N-linked glycosites (localization probability > 0.75), mapping to 436 non-redundant N-glycoproteins (shown in Supplementary Table S1), were quantified from secreted proteins of the three cell lines (SGC7901, SGC7901/ADR and SGC7901/VCR), among which 613 N-glycosites and 286 N-glycoproteins were quantified in all the three cell lines. In addition, we totally identified 2222 N-linked site-specific glycoforms with high confidence. The detailed information of N-linked glycosites, N-glycoproteins and site-specific glycoforms identified from each cell line is summarized in Figure 2.

To verify whether the identified glycoproteins were secretory proteins, we applied bioinformatics softwares and Uniprot database to predict protein secretion pathway. 272 proteins were predicted to be secreted in the classical secretory pathway (138 proteins predicted by SignalP program and 134 proteins including the Uniprot keyword “secreted and/or signal”). In addition, the SecretomeP software presented that 74 proteins were secreted through the nonclassical secretory pathway. In short, these results manifested that 79% of the identified glycoproteins could be released into the conditioned medium by different secretory pathways, demonstrating high efficiency of the platform for analysis of secreted protein glycosylation (Supplementary Figure S1).

To further characterize the identified secreted glycoproteins, protein ontology analysis was executed by Panther software to interpret the functions and involved pathways of the 346 secreted glycoproteins. As shown in Supplementary Figure S2, these glycoproteins had multiple functions and participated in diverse pathways that might play vital roles in the development of MDR. The top three most common molecular functions were catalytic activity (32.8%), binding (27.0%) and receptor activity (25.0%). Besides, the major involved pathways related to MDR were integrin signaling pathway, TGF-β signaling pathway, cadherin signaling pathway, angiogenesis, Wnt signaling pathway and apoptosis signaling pathway.

Then we, for the first time, attempted to illuminate the possible correlations of site-specific glycosylation of glycoproteins with MDR through comprehensively analyzing the changes of glycosites and site-specific glycoforms of secreted proteins during MDR in gastric cancer.

### Characterization of N-glycosite occupancy varying with MDR

**Figure 3:**
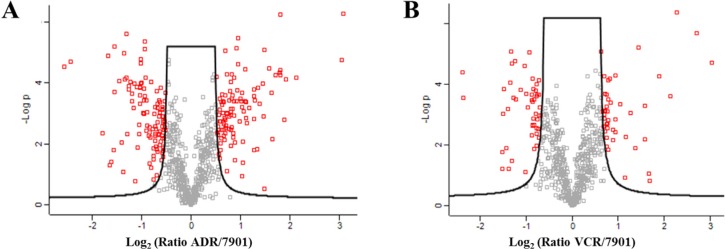
The comparison of glycosites identified in SGC7901 with those identified in two MDR sublines respectively 7901: SGC7901; ADR: SGC7901/ADR; VCR: SGC7901/VCR; and the differentially expressed glycosites with *p*-value ≤ 0.01 are marked red.

Because of significant changes in glycosite occupancy during MDR, the quantification of glycosites was important for studying MDR. We compared the N-glycosites of SGC7901 cell line with those of two drug-resistant cell lines, respectively. About 81 glycosites were only quantified in both of the MDR cell lines, while 75 glycosites were only identified in secreted proteins of SGC7901 (Figure 2A, Supplementary Table S1). Additionally, among the 613 glycosites identified in all the three lines, 84 glycosites in secreted glycoproteins were significantly changed between SGC7901 and the MDR cell lines with *p*-value ≤ 0.01 by *t*-test (Figure 3). Totally, 240 N-glycosites were found dramatically changed in SGC7901-ADR/VCR (124 increased and 116 decreased) compared to SGC7901, as illustrated in Supplementary Table S1. And the significantly different glycosites could be mapped to 163 glycoproteins (84 glycoproteins for up-regulated glycosites, 76 glycoproteins for down-regulated glycosites). Interestingly, there are 3 proteins (IGF2R, ITGB1 and PTPRF) that both have up-regulated and down-regulated glycosites, among which 2 glycoproteins (IGF2R, ITGB1) have been demonstrated to be involved in drug-resistance. Interestingly, as a major player in chemoresistance, ITGB1 (integrin beta-1) could mediate the anti-apoptosis effects of TIMP1 (further discussed below) in melanoma [30].

**Figure 4:**
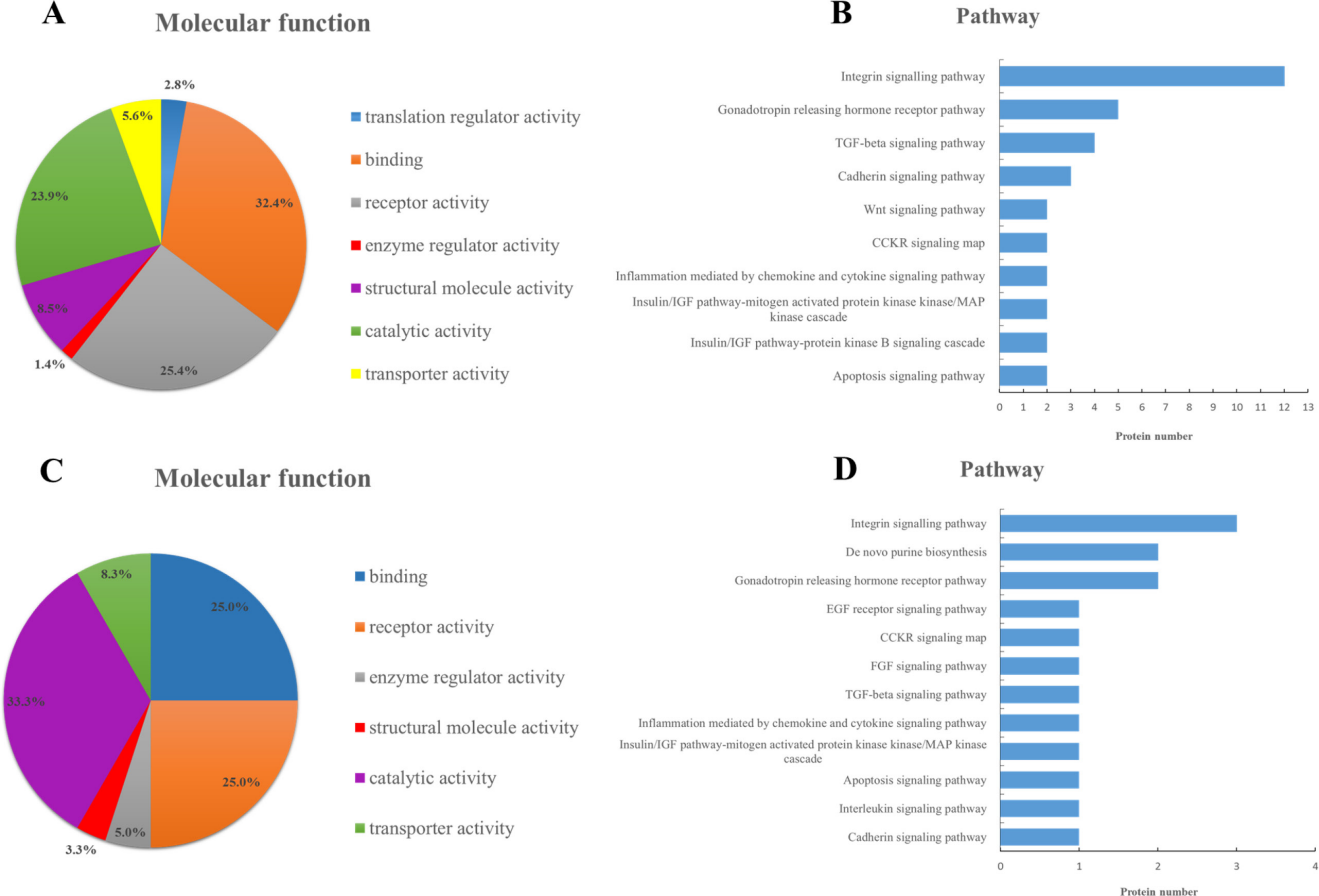
Ontology analysis of 163 glycoproteins corresponding to significantly different glycosites Categories of molecular functions and involved pathways of glycoproteins for increased glycosites (**A**, **B**) and glycoproteins for decreased glycosites (**C**, **D**) in drug-resistant cells.

Further bioinformatics analysis indicated that the leading three molecular functions of 87 glycoproteins for increased glycosites were binding (32.4%), receptor activity (25.4%) and catalytic activity (23.9%), while for 79 glycoproteins corresponding to decreased glycosites, catalytic activity (33.3%), receptor activity (25.0%) and binding (25.0%) ranked the top three. And these three molecular functions were closely related to drug resistance. Moreover, in terms of specific pathways, the numbers of annotated glycoproteins in category of increased glycosites outperformed those in category of decreased glycosites. For example, 12 glycoproteins in Integrin signaling pathway and 4 glycoproteins in TGF-beta signaling pathway were annotated in the cluster of glycoproteins with increased glycosites, much more than those annotated in the cluster of glycoproteins for decreased glycosites. The detailed information was provided in Figure 4. It seemingly highlighted the importance of increased glycosites and their corresponding glycoproteins in the rise of MDR.

### Identification of altered site-specific glycoforms between SGC7901 and its MDR counterparts

Previous studies had demonstrated that N-glycan profiles changed remarkably after chemotherapy in various cancers, and multiple N-glycomic alterations were prominently associated with drug resistance [22, 31]. Ma et al. [31] reported that increased core-fucosylated and sialylated glycans were, to some extent, responsible for MDR in breast cancer cells. However, it was not the same case in MDR of gastric cancer. In the present study, we merely found increased core-fucosylation and hybrid glycans after acquired drug resistance in gastric cancer cell lines, while the other major glycoforms did not show distinct changes. More interestingly, the two MDR sublines exhibited diverse glycomic changes compared to their parental cell line in most cases (Supplementary Table S2). This might be caused by the heterogeneity of glycan structures onto the glycosites. Nevertheless, we maintained that this result could not fully represent the comprehensive glycomic alterations during MDR, as glycosylation contained the information of peptide backbone sequences, glycosite location and glycan structures, simultaneously. Thus, the determination of site-specific glycoforms in secreted proteins could completely reveal the heterogeneity of glycosylation. As shown in Figure 2C, about 31% (678/2222) of the glycoforms were identified in at least two cell lines and the overlap of site-specific glycoforms of three gastric cancer cell lines was merely 12% (272/2222) of the total identified glycoforms, which demonstrated that site-specific glycoforms did dramatically change between drug sensitive and resistant cancer cells. The detailed information of site-specific glycoforms of each cell line was displayed in Supplementary Table S2.

Moreover, we further characterized the significantly different site-specific glycoforms between SGC7901 and its MDR counterparts. As shown in Supplementary Table S2, 175 site-specific glycoforms were increased in secreted proteins of MDR cell lines, while 324 glycoforms were decreased. Interestingly, we discovered that some glycosites contained both up-regulated and down-regulated glycoforms, indicating the high complexity of glycosylation. Totally, 499 site-specific glycoforms onto 151 glycosites in 106 glycoproteins were significantly changed between SGC7901 and its MDR derivates. Further analysis of the mapped glycoproteins showed that most of the annotated glycoproteins were involved in pathways of Integrin, P53, Wnt, Notch, TGF-beta and Hedgehog (Supplementary Figure S3), all of which had been reported to play critical roles in drug resistance of cancer.

### Integrative characterization of changed sites and altered site-specific glycoforms

As described above, both of the glycosites and site-specific glycoforms were greatly different between drug-sensitive and drug-resistant cell lines. Notably, we found that the glycosites with significant changes on glycosite-level rarely intersected with those corresponding to altered site-specific glycoforms (Supplementary Table S3). Moreover, we analyzed the glycosites with significantly altered site-specific glycoforms, and found that about 54% of the glycosites contained more than two significant glycoforms (shown in Supplementary Figure S4), which indicated the high heterogeneity of glycosylation during MDR process. And a total number of 57 glycoproteins with more than 2 significantly different glycoforms onto one specific glycosite were identified (Supplementary Table S4). 33 of the glycoproteins had been reported to be associated with MDR of multiple cancers by searching in PubMed (Table 1). Thus, the quantification of glycosites combined with determination of glycoforms could preferably demonstrate the relationship between changes of glycosylation and MDR. The identified glycoproteins with significant changes on site-level and glycoform-level would be the key points of our further research on chemoresistance in gastric cancer.

**Table 1:** 33 MDR-related glycoproteins with significantly altered glycoforms at one specific site ≥ 2

Uniprot accession number	Protein name	Gene name
P01033	Tissue inhibitor of metalloproteinase 1	TIMP1
P07602	Proactivator polypeptide	PSAP
P11047	Laminin subunit gamma-1	LAMC1
P32004	Neural cell adhesion molecule L1	L1CAM
P10909	Clusterin	CLU
P15328	Folate receptor alpha	FOLR1
H3BMA1	Mesothelin	MSLN
P07996	Thrombospondin-1	THBS1
P98160	Basement membrane-specific heparan sulfate proteoglycan core protein	HSPG2
P80188	Neutrophil gelatinase-associated lipocalin	LCN2
P30530	Tyrosine-protein kinase receptor UFO	AXL
E2D5S3	MHC class I antigen	HLA-C
P05187	Alkaline phosphatase, placental type	ALPP
P07339	Cathepsin D	CTSD
P17936	Insulin-like growth factor binding protein 3	IGFBP3
D6W5P7	ADAM metallopeptidase domain 22	ADAM22
P50897	Palmitoyl-protein thioesterase 1	PPT1
Q13740	CD166 antigen	ALCAM
Q92820	Gamma-glutamyl hydrolase	GGH
P41271	Neuroblastoma suppressor of tumorigenicity 1	NBL1
B9EJB8	Collagen, type XII, alpha 1	COL12A1
P07942	Laminin subunit beta-1	LAMB1
P51884	Lumican	LUM
O94907	Dickkopf-related protein 1	DKK1
Q03405	Urokinase plasminogen activator surface receptor	PLAUR
P56199	Integrin alpha-1	ITGA1
Q9BY76	Angiopoietin-related protein 4	ANGPTL4
P02750	Leucine-rich alpha-2-glycoprotein	LRG1
C9JBB3	Tissue factor pathway inhibitor	TFPI
M0QZZ9	Mucin-16	MUC16
P28799	Granulins	GRN
Q54A51	Basigin (Ok blood group), isoform CRA_a	hEMMPRIN
P11717	Insulin-like growth factor 2 receptor	IGF2R

## DISCUSSION

In the current study, we provided the most comprehensive secretome glycoproteomic characterization of SGC7901 and its MDR counterparts SGC7901/ADR, SGC7901/VCR. These three gastric cancer cell lines are extensively and intensively studied in our lab to elucidate the underlying mechanism of MDR in GC using genomic and proteomic approaches [32, 33]. However, these studies mainly focused on the protein expression, but provided little information about protein glycosylation.

Herein, for the first time, we explored the correlations between glycosylation, particularly site-specific glycosylation, in secreted glycoproteins and multidrug resistance in gastric cancer. Totally, 1033 N-glycosites in 436 glycoproteins and 2222 N-linked site-specific glycoforms were identified and quantified from secreted proteins of SGC7901 and its MDR counterparts. It was noteworthy that core-fucosylation was increased in MDR cells. And, others had reported similar results in breast cancer cells [31] and prostate cancer cells [28]. Moreover, overexpression of two fucosyltransferases (FUT8 and FUT11) was probably responsible for the change of fucosylation observed in MDR cells [28]. Cheng et al. demonstrated that the alterations of FUTs (FUT4, FUT6 and FUT8) were involved in MDR in human hepatocellular carcinoma cells by modulating PI3K/Akt signaling pathway and MRP1 expression [34]. In our study, the increased fucosylation may be due to the up-regulation of FUT8 and FUT11 based on our mRNA sequencing results (data not shown). But, this hypothesis needs to be validated by further studies.

Besides, our preliminary results manifested that glycosites and site-specific glycoforms of secreted glycoproteins in MDR cells vastly differed from those in the parental ones. Compared to SGC7901, about 499 site-specific glycoforms in 106 glycoproteins were significantly changed with the similar trend in the two MDR cell lines. We further identified 57 glycoproteins with more than 2 significantly different site-specific glycoforms at one specific glycosite between drug-sensitive and resistant cell lines, and found that more than half of them had been demonstrated to be involved in chemoresistance of various cancer types, among which the key roles of AXL, L1CAM, TIMP1 and Clusterin in MDR were massively studied. Yet, there are few reports about the relationship between glycosylation of secretory proteins and MDR.

**Figure 5:**
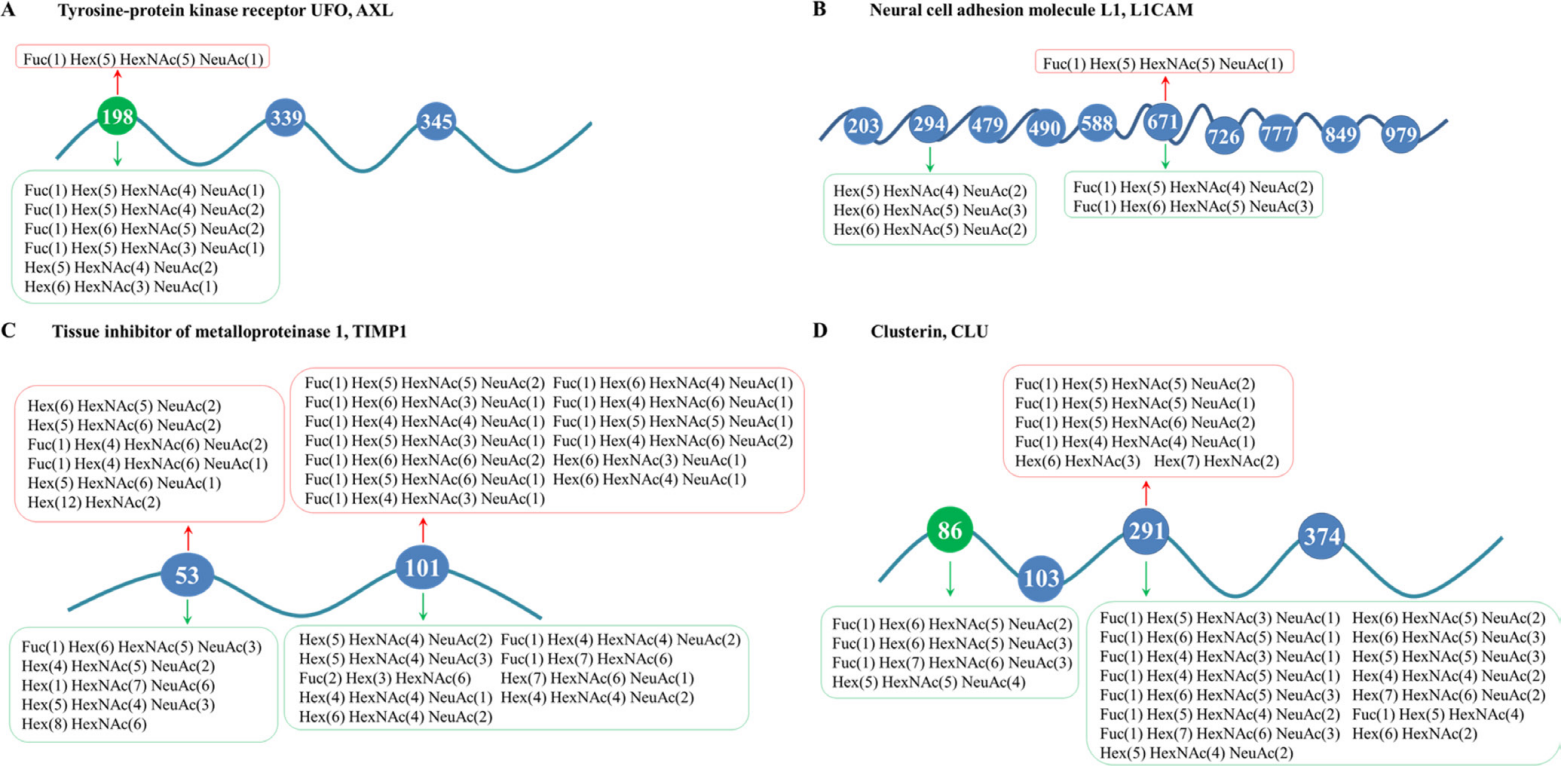
Schemes for changes of glycosites and site-specific N-glycosylation in MDR cells of (A) AXL; (B) L1CAM; (C) TIMP1; and (D) CLU N-glycans that were up-regulated in MDR cells were placed above the peptides and marked red, while those down-regulated in MDR cells were put under the peptides and marked green. Besides, glycosites significantly decreased in MDR cells were marked green.

**Figure 6:**
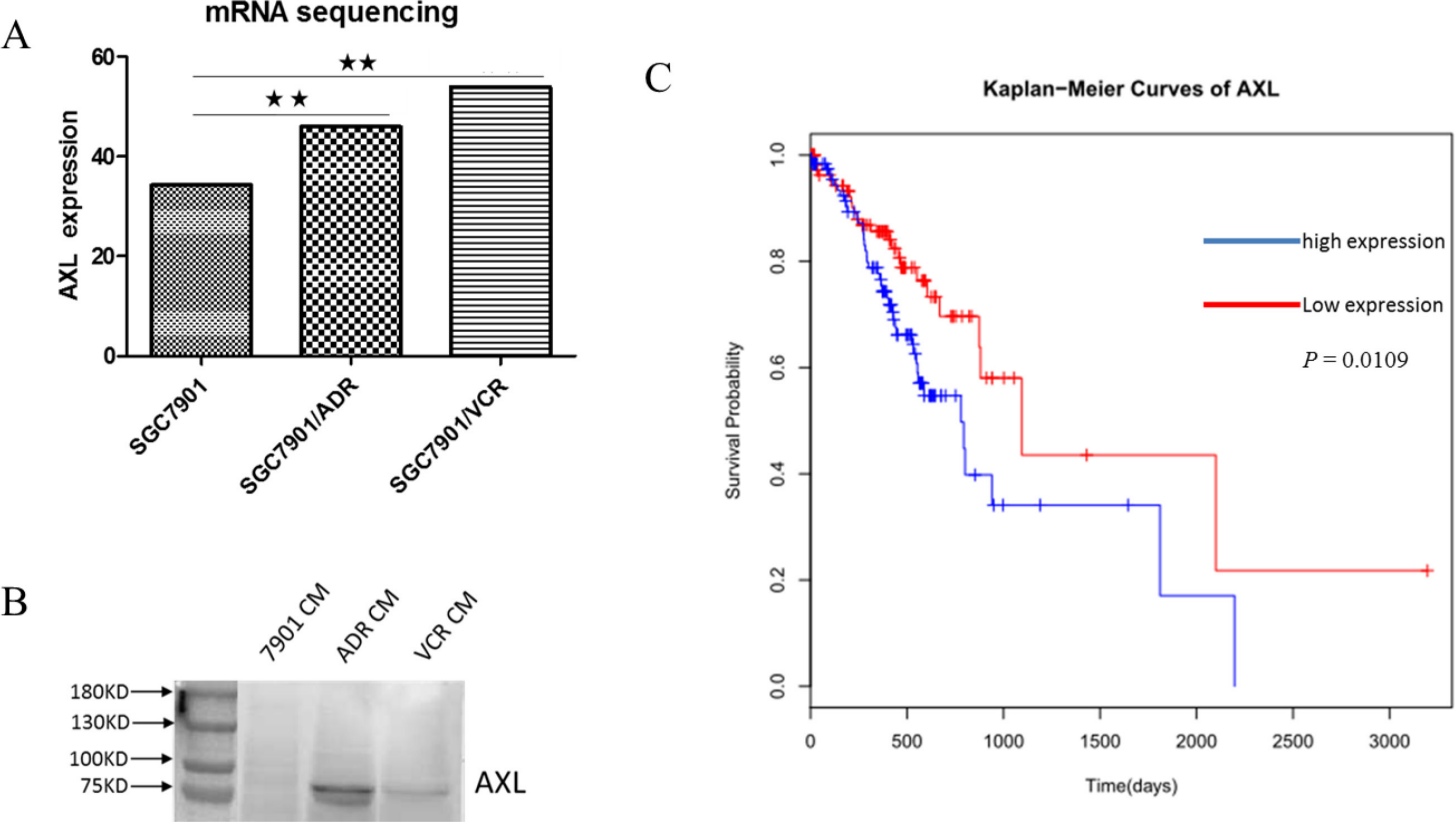
AXL is increased in GC MDR cells and predicts poor survival in GC patients (**A**) The mRNA expression of AXL is upregulated in GC MDR cells. *P* < 0.01. (**B**) The protein expression of AXL is increased in the conditioned medium of GC MDR cells. CM: conditioned medium. C. Based on TCGA database, high expression of tumor AXL predicts worse survival of GC patients.

Here, we would comprehensively discuss the changes of glycosylation of four candidate glycoproteins during MDR by integrative analysis of glycosites and site-specific glycoforms. The receptor tyrosine kinase AXL is over-expressed in different types of cancers and implicated in several malignant phenotypes of tumor cells, such as invasion and chemoresistance [35, 36]. AXL could mediate multi-drug resistance by modulation of microRNAs and EMT (epithelial-to-mesenchymal transition), as well as signaling pathways of PI3K/Akt and EGFR/PKC/mTOR [37–41]. More importantly, aberrant glycosylation of AXL was reported to contribute to tumor proliferation, invasion and metastasis [42], and alpha-2, 6-sialyltransferases ST6GalNAcII was further identified as critical modulator of its functions in mammary phyllodes tumors [43]. In this work, we characterized the changes of site-specific glycosylation in secretory AXL during the acquisition of MDR in stomach cancer for the first time. Generally speaking, we identified three N-glycosites – Asn198, Asn339 and Asn345, among which Asn198 was found significantly reduced in MDR cells. Intriguingly, seven significantly differentially expressed glycoforms were quantified at Asn198 and the other two glycosites were identified without distinctly altered N-glycans (Figure 5A, Supplementary Table S5), which possibly implied the importance of Asn198 in the actions of AXL. Soluble AXL may result from the proteolytic cleavage of receptor tyrosine kinase AXL [44]. It was reported that soluble AXL could outperform AFP in diagnosing very early hepatocellular carcinoma [45] and correlated with tumor stage as well as patient survival in renal cell carcinoma [46]. While, our further work validated the increased mRNA expression of AXL in GC MDR cells and increased protein expression of soluble AXL in the conditioned medium of GC MDR cells. In addition, we found that high expression of tumor AXL predicted worse survival in GC patients based on TCGA database (Figure 6). In a word, glycosylation and expression differences of soluble AXL between GC drug-sensitive and resistant cells have been observed, suggesting a significant role of soluble AXL in the MDR of GC, but this hypothesis requires further researches to explore how soluble AXL and aberrantly glycosylated AXL influence the MDR of GC.

We also characterized the site-specific glycosylation changes of three other proteins—L1CAM, TIMP1 and CLU, during MDR of GC cells (Figure 5B–5D, Supplementary Tables S6–S8). These proteins are all heavily glycosylated proteins and known to be involved in resistance to chemotherapies in multiple cancer types [47–52], [16, 53, 54]. Moreover, as glycoproteins, the functions of TIMP1 and CLU were reported to be markedly influenced by altered glycans [55–57]. Nevertheless, previous studies rarely focused on the alterations of protein glycosylation during multi-drug resistance in cancer. In our study, we identified and quantified the differences of glycosites and site-specific glycoforms of L1CAM, TIMP1 and CLU between SGC7901 and its MDR derivates, and found significant changes on glycosites as well as site-specific glycans. This suggested that glycosylation at specific glycosites might possibly have an enormous impact on actions of the three proteins in MDR of gastric cancer. Further researches are needed to confirm this plausible conclusion.

In conclusion, our present work could well characterize the site-specific glycosylation of secreted glycoproteins, including the glycosite occupancy as well as glycan structures. And, we believe that glycosylation at specific positions and its changes in secreted glycoproteins would be greatly involved in the acquisition of MDR, and our work could provide new insights into the sophisticated mechanisms underlying MDR in gastric cancer. Furthermore, the aberrantly glycosylated secreted glycoproteins obtained in our work would be candidate biomarkers for MDR prediction or key targets to reverse drug resistance in gastric cancer. Further investigations are warranted to verify this.

## MATERIALS AND METHODS

### Reagents and materials

Trypsin, 1, 4-dithiothreitol (DTT), iodacetamide (IAA), trifluoroacetic acid (TFA) and formic acid (FA) were obtained from Sigma (St. Louis, MO, USA). PNGase F was purchased from New England Biolabs (Ipswich, MA, USA). The centrifugal filter units (Amicon Ultra, 3 KD, 15 mL) were purchased from Millipore (Milford, MA, USA). Acetonitrile (ACN, HPLC grade) was from Merck (Darmstadt, Germany). Water used in all experiments was purified with a Milli-Q system (Millipore, Milford, MA, USA). C18 AQ beads (3 μm, 120 Å) were from Michrom BioResources (Auburn, CA, USA). The click maltose-HILIC beads were obtained from Xinmiao Liang group (liangxm@dicp.ac.cn).

### Cell culture and secretome collection

Human gastric adenocarcinoma cell line SGC7901 was obtained from the Academy of Military Medical Science (Beijing, China). And the MDR sublines, SGC7901/VCR (VCR) and SGC7901/ADR (ADR), were developed by stepwise selection with vincristine and adriamycin, respectively. The above three cell lines were maintained in RPMI-1640 medium supplemented with 10% fetal bovine serum (FBS), 100 U/ml penicillin sodium and 100 mg/ml streptomycin sulphate at 37°C in a humidified atmosphere containing 5% CO_2_. Vincristine (1 μg/ml) or adriamycin (0.5 μg/ml) was added to the culture medium of corresponding MDR cell sublines to maintain their MDR phenotype. The gastric cancer cell lines were appropriately cultured until ~70% confluence (150 mm cell culture dish). In order to eliminate the possible interference of residual FBS, the cancer cells were washed with PBS (phosphate buffered saline) and serum-free RPMI-1640 medium, respectively. Then, cells were maintained in 30 ml serum-free RPMI-1640 medium at 37°C for another 24 h. The conditioned medium was collected and centrifuged at 1000 g to remove cell debris. Then the secreted proteins samples were concentrated using 3 KD MWCO Ultra Centrifugal Filters. The secreted protein concentration was determined by BCA assay.

### Protein digestion and glycopeptide enrichment

The protein samples were prepared following the previously reported with some modifications. Briefly, 0.8 mg secreted proteins were reduced by DTT at 37°C for 2 h and alkylated by IAA in dark at room temperature for 30 min. Then, trypsin was added with ratio at 1:50 (trypsin/proteins, w/w), and the mix was incubated at 37°C overnight. The trypsin was again added at the same weight ratio, and incubated at 37°C for another 4 h. The tryptic digests were desalted by SPE column (Waters, USA) and dried for further analysis.

The glycopeptide enrichment was performed with click maltose-HILIC materials. The prepared materials (10 mg) were washed by 80% ACN/1% TFA, and the digestions of secreted proteins (160 μg) were mixed with HILIC materials. After incubated at room temperature for 90 min, the supernatant was removed by centrifugation at 20, 000 g for 3 min, and the HILIC materials were further washed with 200 μL 80% ACN/1% TFA for three times. Finally, the glycopeptides were eluted by using 100 μL 30% ACN/1% FA solution for twice, and the eluted fractions were combined. The enriched glycopeptides were divided into two portions, and one part of glycopeptides was deglycosylated by using PNGase F (500 units) in 10 mM NH_4_HCO_3_ (pH 8.0) at 37°C overnight. Then, the enriched glycopeptides and de-glycopeptides from about 50 μg tryptic digestions of proteins were dried for MS analysis, respectively.

### Mass spectrometric analysis

The LC-MS/MS analyses were performed on a Q-Exactive mass spectrometer (Thermo, San Jose, CA) equipped with an Ultimate 3000 system (Thermo, San Jose, CA) for separation. The LC-MS/MS system contained a C18 capillary trap column (200 μm i.d., C18 AQ beads (5 μm, 120 Å)) and a 12-cm C18 capillary analysis column (75 μm i.d., C18 AQ beads (3 μm, 120 Å)). Mobile phases A (98% H_2_O/2% acetonitrile/0.1% FA) and B (80% acetonitrile/20% H_2_O/0.1% FA) were used to develop a gradient. For both glycopeptides and de-glycopeptides, the RP gradient was developed at 300 nl/min as follows: loading sample for 15 min, from 4 to 45% buffer B for 135 min, from 45 to 90% buffer B for 15 min, from 90 to 90% buffer B for 10 min, from 90 to 4% buffer B for 4 min and from 4 to 4% buffer B for 11 min. The temperature of the ion transfer capillary was 220°C and the normalized collision energy was set to 25%. The mass resolution was set to 70, 000 for full MS and 17, 500 for HCD MS/MS. Survey full scan MS was acquired from m/z 400 to 2, 000, and 15 most intense ions were selected for MS/MS scan. The dynamic exclusion was set as follows: repeat count, 1; duration, 30 s; exclusion list size, 500; exclusion duration, 90 s. All the samples, including intact glycopeptides and de-glycopeptides were analyzed for three runs.

### Mass spectrometry data analysis

For glycosite analysis of secreted proteins, the mass spectrometric data of de-glycopeptides acquired by Q-Exactive MS was searched and quantified by using Maxquant (1.5.2.8) [58]. The following parameters were used for searching: mass tolerances were 20 ppm and 0.1 Da for the precursor and fragments, respectively; for trypsin digested samples, enzyme specificity was set to KR/P with up to 2 missed sites; cysteine residue was set as a static modification of 57.0215 Da; Asn deamidation (+0.9840 Da) and methionine oxidation (+15.9949 Da) were set as variable modifications. The glycosites were identified with the motif of N-X (Not Proline)-S/T. For the quantification of glycosites, label free quantification (LFQ) in Maxquant was employed to extract the intensity of full MS, and quantification data was further analyzed by Perseus software (version 1.5.1.6) with Log2 transform and Z-score normalization. The determination of site-specific glycoforms was performed by using ArMone proteomics data processing platform (http://www.bioanalysis.dicp.ac.cn/proteomics/software/ArMone2.html) with some modifications [59]. First, the peptide sequence was identified using Mascot (2.3.0) following the above parameters with FDR for peptide identification of both peptides and proteins < 1%. And information of N-glycosites and peptide sequences was integrated into a format file “Peptide list” (.ppl), while the mass spectrometric data of intact glycopeptide acquired by Q-Exactive MS was converted into format of ‘MZ.xml’ using MS Convert. Then the ‘.ppl’ (deglycopeptides) and ‘MZ.xml’ (intact glycopeptides) were loaded into Armone proteomics data processing platform. The software could automatically extract MS/MS spectra of intact glycopeptides based on oxonium ions, and the Y1 ions (peptide sequence +GlcNAc) could be determined by the characters of N-linked five core glycans. Then the molecular weight of both peptide backbone and glycans could be calculated. And the peptide back bone sequences were determined by matching the MW of Y1 ions to that from de-glycopeptides identified from Mascot search. The backbone sequences were filtered based on the maximum of 15 min in retention time and 20 ppm for MW. The glycan structures were determined by using MW and fragments of specific sugars. The site-specific glycoforms could be determined by combining the two part information, and the results were further filtered with glycopeptide score more than 10, and most of the glycan structures were further manually corrected. For the quantification of site-specific glycoforms, the intensity of glycoforms was extracted by house software, and the intensity was transformed to Log2 and normalized with Z-score by using Perseus software (version 1.5.1.6). Additionally, for glycoforms quantified in the secreted proteins of three cell lines, those meeting the criteria (Ratio MDR/7901 > 2 OR < 0.5) were identified as significantly changed glycoforms. While, the glycoforms determined only in MDR cell lines (ADR&VCR, spectra count > 2) or only in SGC7901 cell line (spectra count > 5) were also judged as significantly changed glycoforms.

Furthermore, the secretome was defined to proteins with the Uniprot keyword “signal or secreted” and those predicted by SignalP 4.1 [60] or SecretomeP 2.0 [61]. In addition, functional classification of the identified glycoproteins was performed by PANTHER classification system [62].

### TCGA data analysis

The clinical and gene expression data of 295 primary gastric adenocarcinomas were derived from TCGA data portal (https://tcga-data.nci.nih.gov/tcga/) and TCGA-based publication [63]. The expression of AXL was divided into high and low expression group by median expression. Then, the association between AXL expression and patient survival was analyzed using the Kaplan-Meier method with the log-rank test in Cox proportional hazards model.

### Western blot

The concentrated secreted protein samples collected above were resolved by 12% SDS-PAGE (Bio-Rad Laboratories, Hercules, CA, USA) and blotted onto nitrocellulose membrane (Amersham Biosciences, Pittsburgh, PA, USA). Membrane was blocked with 10% non-fat milk at room temperature for 2 h and incubated overnight with primary antibody: anti-AXL (1:1000; Abcam, Cambridge, MA, USA). After three 5 min washes in TBST (Triethanolamine-Buffered Saline Solution with 0.1% Tween-20), membrane was incubated with horseradish peroxidase (HRP) conjugated secondary antibody (1:2000; Santa cruz Biotechnology, Dallas, TX, USA) for 2 h at room temperature and then washed again in TBST and visualized with an enhanced chemi-luminescence kit (ECL-kit, santa cruz biotechnology, Dallas, TX, USA). All experiments were performed in triplicate.

## SUPPLEMENTARY MATERIALS FIGURES AND TABLES


















