# Supplementary material for: Characterization of site-specific glycosylation of secreted proteins associated with multi-drug resistance of gastric cancer
Source: Oncotarget. 2016 Mar 23;7(18):25315–27. doi: 10.18632/oncotarget.8287 (PMC5041906; doi:10.18632/oncotarget.8287)
Supplement: Supplementary file 1 [file oncotarget-07-25315-s001.pdf]

Characterization of site-specific glycosylation of secreted proteins associated with multi-drug resistance of gastric cancer

Supplementary Materials

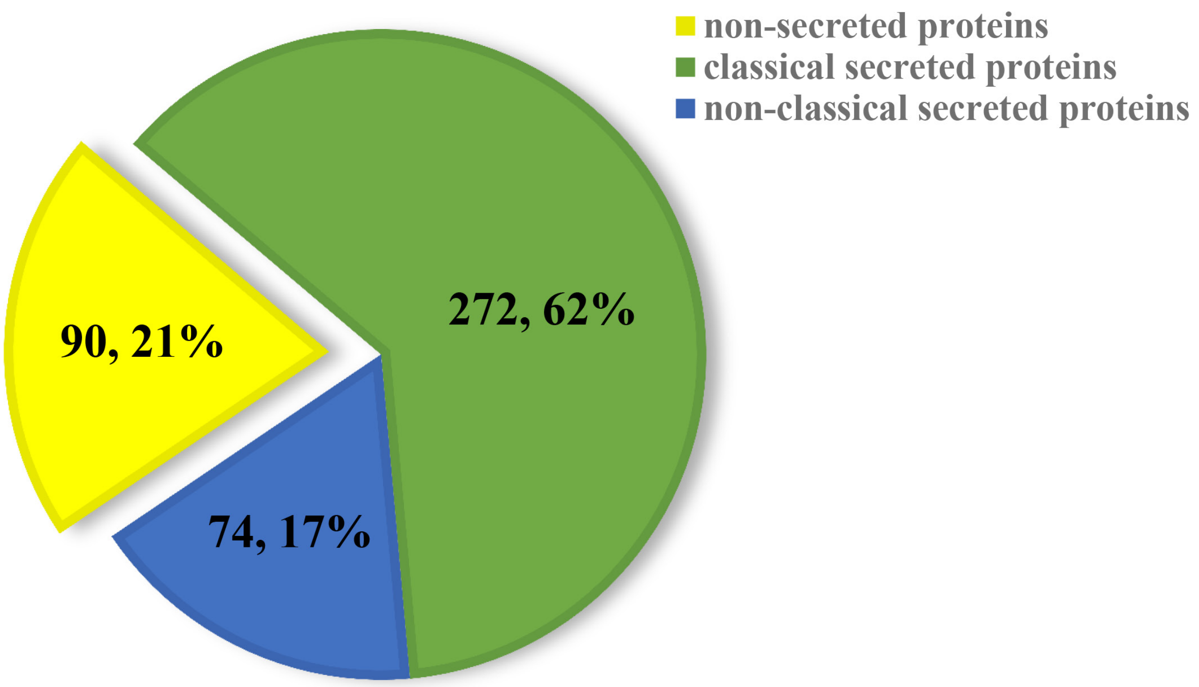

Supplementary Figure S1: The validation of secreted glycoproteins in total identified glycoproteins. (number, percentage).

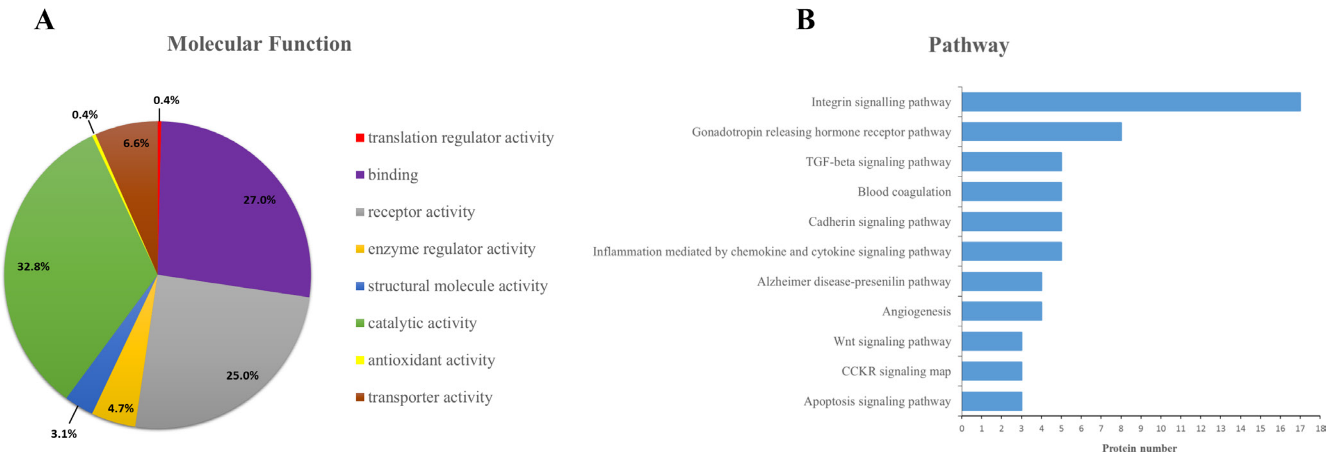

Supplementary Figure S2: Classification of the identified secreted glycoproteins by Panther. A for category of molecular function and B for category of involved pathway.

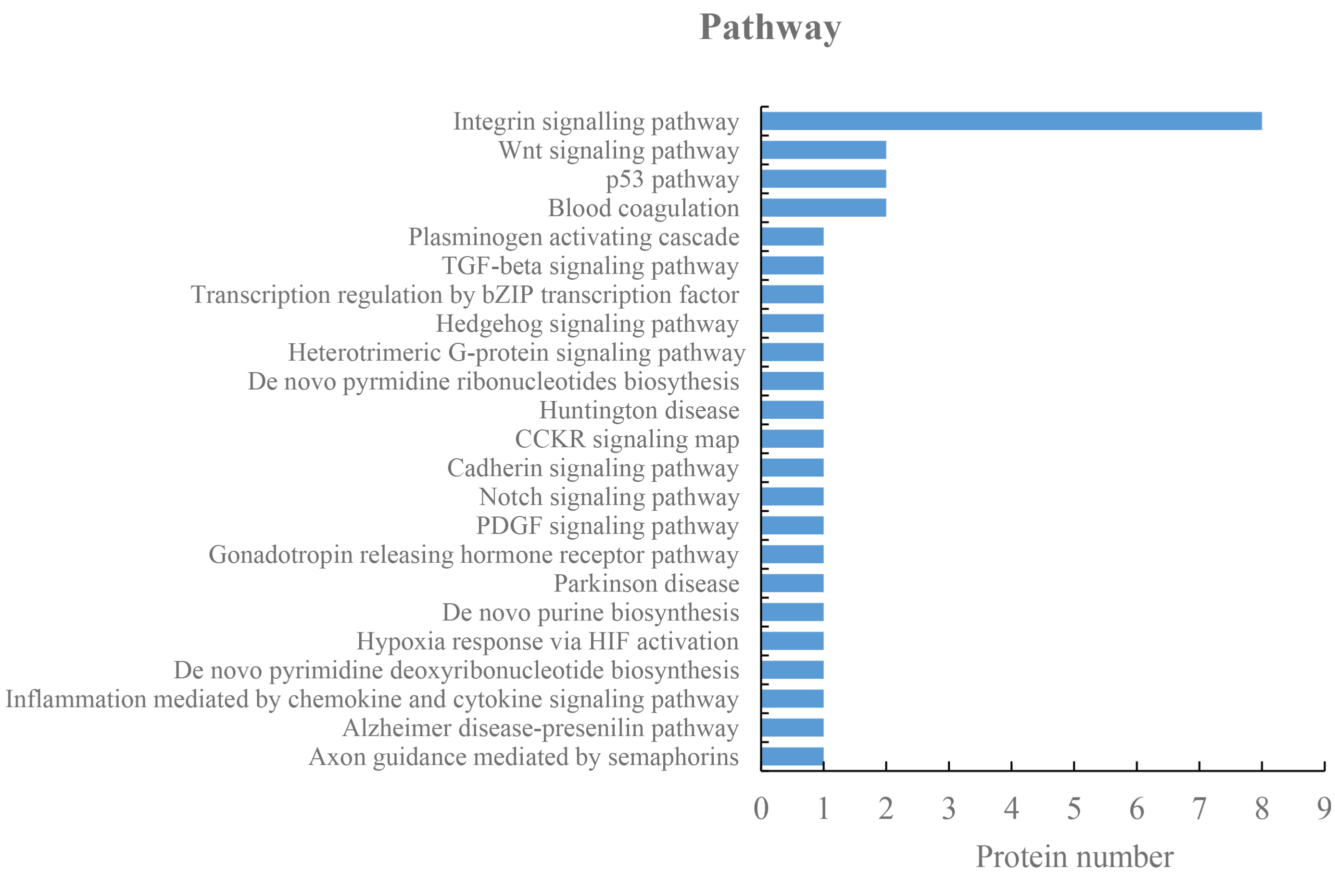

Supplementary Figure S3: Pathway analysis of 106 glycoproteins corresponding to significantly different site-specific glycoforms between drug-sensitive and drug-resistant cells.

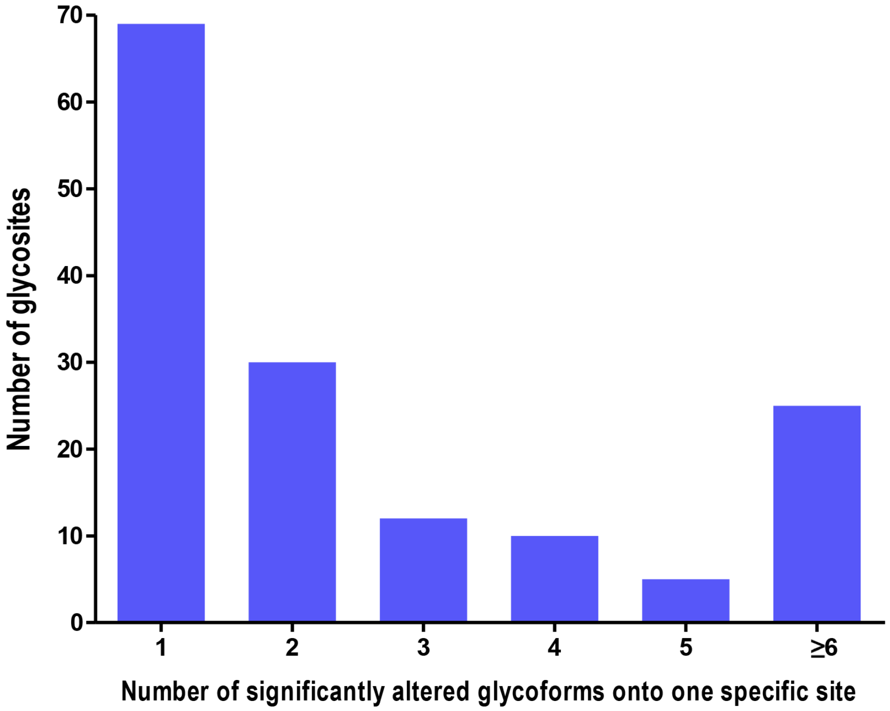

Supplementary Figure S4: The distribution of glycosites corresponding to significantly different site-specific glycoforms.

Supplementary Table S1: MS information of glycosites

Supplementary Table S2: MS information of site-specific glycoforms

Supplementary Table S3: Overlap of identified glycosites on glycosite level and glycoform level

Supplementary Table S4: The glycosites corresponding to significantly different glycoforms

Supplementary Table S5: AXL

Supplementary Table S6: L1CAM

Supplementary Table S7: TIMP1

Supplementary Table S8: Clusterin
